# Long-term prognostic value of thyroid hormones in left ventricular noncompaction

**DOI:** 10.1007/s40618-024-02311-8

**Published:** 2024-02-15

**Authors:** L. Liu, S. Cai, A. Chen, Y. Dong, L. Zhou, L. Li, Z. Zhang, Z. Hu, Z. Zhang, Y. Xiong, Z. Hu, Y. Li, M. Lu, L. Wu, L. Zheng, L. Ding, X. Fan, Y. Yao

**Affiliations:** 1 Cardiac Arrhythmia Center, Fuwai Hospital, State Key Laboratory of Cardiovascular Disease, National Center for Cardiovascular Diseases, National Clinical Research Center for Cardiovascular Diseases, Chinese Academy of Medical Sciences and Peking Union Medical College, 167 Beilishi Road, Xicheng District, Beijing, 100037 China; 2 Cardiac Arrhythmia Center, Heart Center, The People’s Hospital of Zhengzhou University, Henan Provincial People’s Hospital, Huazhong Fuwai Hospital, Zhengzhou, Henan China; 3 Department of Echocardiography, Fuwai Hospital, State Key Laboratory of Cardiovascular Disease, National Center for Cardiovascular Diseases, Chinese Academy of Medical Sciences and Peking Union Medical College, Beijing, China; 4 Department of Magnetic Resonance Imaging, Fuwai Hospital, State Key Laboratory of Cardiovascular Disease, National Center for Cardiovascular Diseases, Chinese Academy of Medical Sciences and Peking Union Medical College, Beijing, China

**Keywords:** Thyroid function, Free triiodothyronine, Left ventricular noncompaction, Mortality, Major adverse cardiovascular event

## Abstract

**Purpose:**

Thyroid function is closely related to the prognosis of cardiovascular diseases. This study aimed to explore the predictive value of thyroid hormones for adverse cardiovascular outcomes in left ventricular noncompaction (LVNC).

**Methods:**

This longitudinal cohort study enrolled 388 consecutive LVNC patients with complete thyroid function profiles and comprehensive cardiovascular assessment. Potential predictors for adverse outcomes were thoroughly evaluated.

**Results:**

Over a median follow-up of 5.22 years, primary outcome (the combination of cardiovascular mortality and heart transplantation) occurred in 98 (25.3%) patients. For secondary outcomes, 75 (19.3%) patients died and 130 (33.5%) patients experienced major adverse cardiovascular events (MACE). Multivariable Cox analysis identified that free triiodothyronine (FT3) was independently associated with both primary (HR 0.455, 95%CI 0.313–0.664) and secondary (HR 0.547, 95%CI 0.349–0.858; HR 0.663, 95%CI 0.475–0.925) outcomes. Restricted cubic spline analysis illustrated that the risk for adverse outcomes increased significantly with the decline of serum FT3. The LVNC cohort was further stratified according to tertiles of FT3 levels. Individuals with lower FT3 levels in the tertile 1 group suffered from severe cardiac dysfunction and remodeling, resulting in higher incidence of mortality and MACE (Log-rank *P* < 0.001). Subgroup analysis revealed that lower concentration of FT3 was linked to worse prognosis, particularly for patients with left atrial diameter ≥ 40 mm or left ventricular ejection fraction ≤ 35%. Adding FT3 to the pre-existing risk score for MACE in LVNC improved its predictive performance.

**Conclusion:**

Through the long-term investigation on a large LVNC cohort, we demonstrated that low FT3 level was an independent predictor for adverse cardiovascular outcomes.

**Graphical Abstract:**

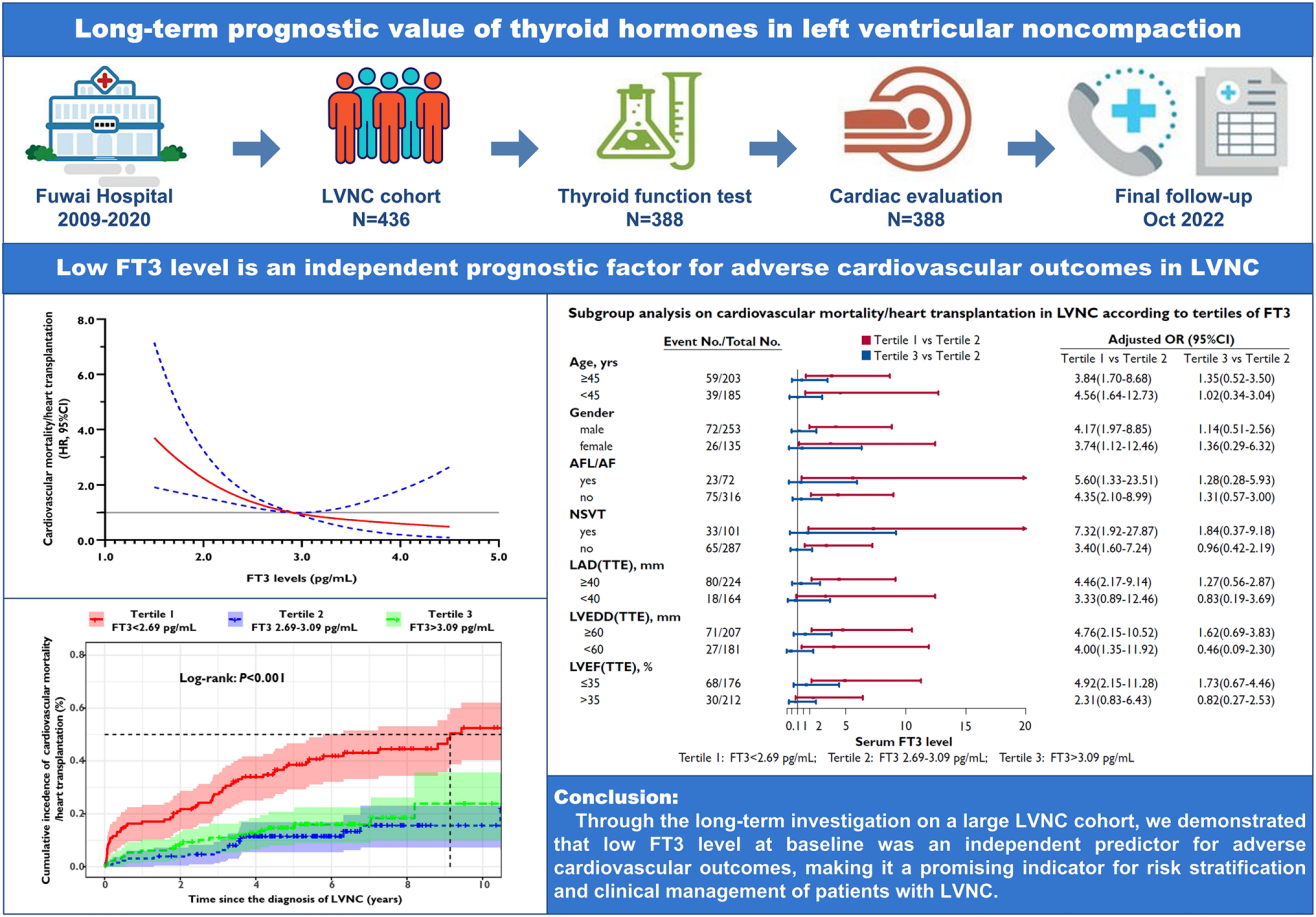

**Supplementary Information:**

The online version contains supplementary material available at 10.1007/s40618-024-02311-8.

## Introduction

Thyroid hormones (THs) have far-reaching and profound effects on the cardiovascular system. They act as fundamental regulators in cardiovascular homeostasis by impacting cardiac contractility, blood pressure, vascular resistance, and heart rhythm [1, 2]. Given that the action of THs is mediated by specific triiodothyronine (T3) receptors in the myocardium, the physiological function of heart predominantly relies on the biologically active free T3 (FT3). Reduced levels of FT3 may impair myocardial contractility, lead to ventricular remodeling, increase susceptibility to arrhythmias, and contribute to mortality in cardiac failure [3, 4]. A growing body of clinical and experimental evidence has suggested that decreased FT3 level is a crucial prognostic factor for a range of cardiac disorders including heart failure, cardiomyopathy, and coronary artery disease [5–11].

Left ventricular noncompaction (LVNC) is a heterogeneous myocardial disorder characterized by excessive trabeculations and intertrabecular recesses connected to the left ventricle, giving the myocardium a bilayered appearance of thick noncompacted and thin compacted structure [12]. It has been recognized as a relatively rare distinct cardiomyopathy [13], with various clinical manifestations ranging from asymptomatic to severe conditions such as congestive heart failure, malignant ventricular arrhythmias, systemic embolisms, and even sudden cardiac death (SCD) [14, 15]. The pathogenesis of LVNC was traditionally attributed to an arrest in the compaction process of myocardium during embryonic stage [12], while recent studies have suggested that mutations in genes encoding sarcomere, cytoskeletal or ion channel proteins might be related to the development of the disease [16, 17]. Despite a number of existing definitions, the current approach to diagnose LVNC mainly relies on the ratio of noncompacted to compacted myocardium, and the most commonly used diagnostic criteria for adult LVNC patients are the Jenni criteria via transthoracic echocardiogram (TTE) and Petersen criteria via cardiac magnetic resonance (CMR) [18, 19]. Due to the remarkable heterogeneity and wide spectrum of clinical presentations, the long-term prognosis of LVNC remains incompletely elucidated, and clinical evidence regarding its risk stratification is rather limited. Even though the predictive value of THs has been observed in several cardiovascular conditions, there is currently a lack of literature addressing the connection between THs and prognosis of LVNC. Therefore, we investigated the long-term follow-up data from a large cohort of morphologically diagnosed LVNC patients, and sought to explore the association between THs and adverse cardiovascular outcomes in this particular population.

## Materials and methods

### Study design and population

This was an observational, retrospective, longitudinal cohort study that followed the Strengthening the Reporting of Observational Studies in Epidemiology (STROBE) reporting guideline [20]. Patients who were diagnosed with LVNC according to the Jenni criteria by TTE and/or the Petersen criteria by CMR at Fuwai Hospital between January 1, 2009 and December 31, 2020 were consecutively recruited. Each individual underwent a thorough medical record review and a comprehensive cardiovascular assessment at baseline. Patients were further followed through telephone interviews and/or outpatient visits, and the final follow-up occurred in October 2022.

**Fig. 1:**
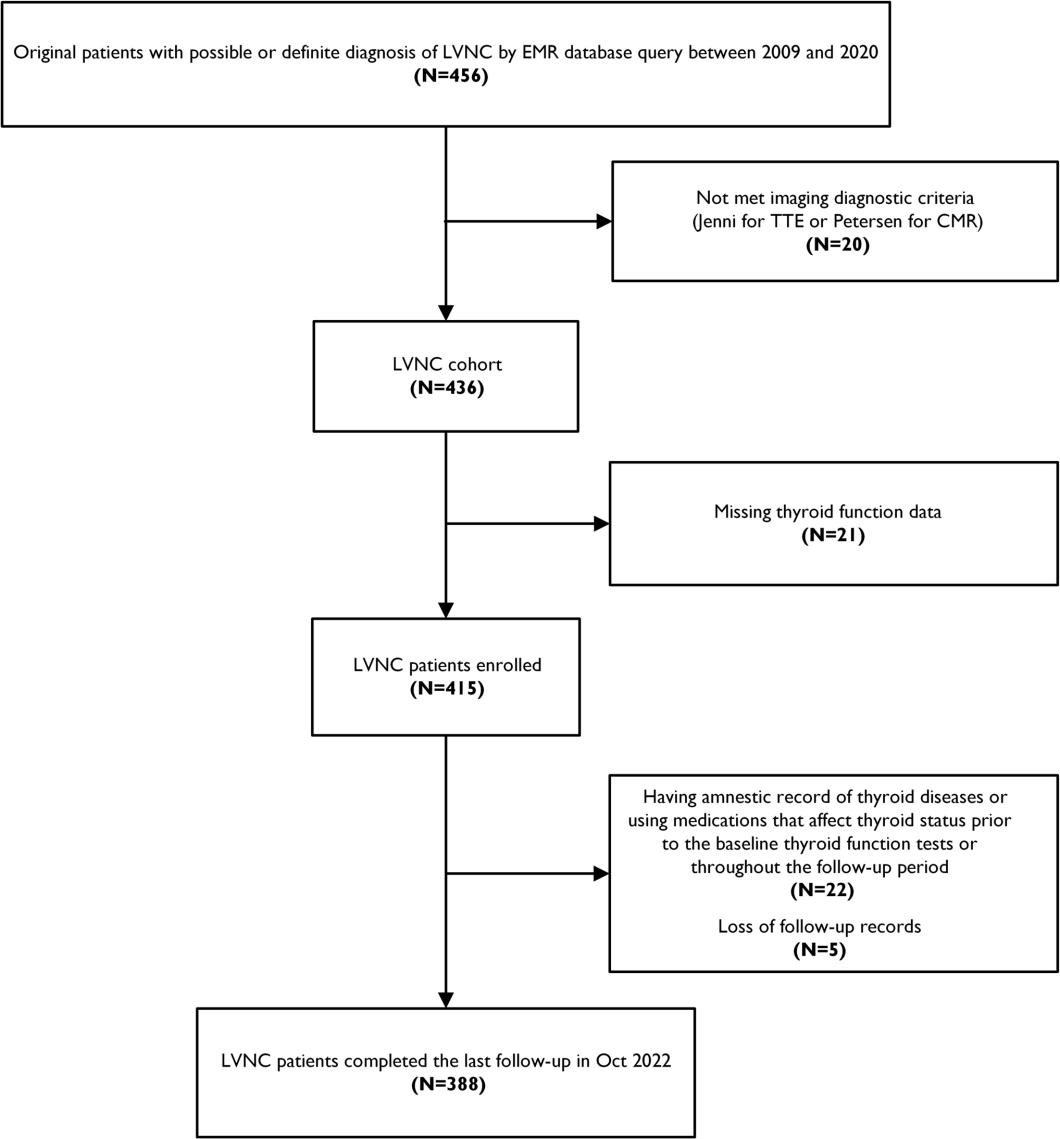
Flow chart of patient inclusion and exclusion criteria. LVNC, left ventricular noncompaction; EMR, electronic medical record; TTE, transthoracic echocardiography; and CMR, cardiac magnetic resonance

Out of 456 original patients with possible diagnosis of LVNC through the medical record database query, 20 failed to meet the imaging diagnostic criteria for the disease. We further excluded participants who (i) lacked baseline data for thyroid function tests; (ii) had an amnestic record of thyroid diseases or reported use of medications that might affect thyroid status (including thyroid hormone replacement, antithyroid drugs, amiodarone, corticosteroids, etc.) prior to the baseline thyroid function tests or throughout the patients’ follow-up, and (iii) were lost to follow-up and unable to provide details on the occurrence and date of clinical outcomes. Ultimately, a total of 388 eligible LVNC patients were included in the present study (Fig. 1).

### Laboratory measurement of thyroid function

Blood samples were taken from patients after fasting for twelve hours, and thyroid function was detected at baseline. The serum levels of FT3, total triiodothyronine (TT3), free thyroxine (FT4), total thyroxine (TT4), and thyrotropin (TSH) were measured using radioimmunoassay (Immulite 2000, Siemens, Germany). The following showed the reference ranges of THs in our laboratory: FT3, 1.79–4.09 pg/mL; TT3, 0.65–1.91 ng/mL; FT4, 0.80–1.88 ng/dL; TT4, 4.29–12.47 mg/dL; and TSH, 0.55–4.78 mIU/L.

### Cardiac evaluation

Examinations of TTE were conducted using a Phillips iE33 Color Doppler Ultrasound System (Philips Healthcare, Andover, MA). Two-dimensional and M-mode images of left atrial diameter (LAD) and left ventricular end-diastolic diameter (LVEDD) were recorded from the parasternal long-axis acoustic window. Left ventricular ejection fraction (LVEF) was calculated using the modified biplane Simpson's rule. CMR studies were performed using a 1.5-T scanner (Magnetom Avanto, Siemens Medical Solutions, Erlangen, Germany). Cine images in LV long-axis, LV short-axis, and horizontal long-axis orientations were obtained through true fast imaging with a steady-state precession sequence. Images of late gadolinium enhancement (LGE) were acquired 10–15 min after a bolus injection of 0.2 mmol/kg gadolinium-diethylenetriamine pentaacetic acid (Magnevist, Schering AG, Berlin, Germany). Cardiac thrombosis was diagnosed if any presence of thrombus was detected in the cardiac cavity by TTE and/or CMR. The imaging diagnostic criteria for LVNC were based on the Jenni criteria by TTE and/or the Petersen criteria by CMR, with CMR taking precedence over TTE in case of discrepancy. Jenni criteria were defined as any segment of ventricular myocardium with maximum end-systolic noncompacted: compacted thickness ≥ 2 [18], whereas Petersen criteria were defined as end diastolic noncompacted: compacted thickness ≥ 2.3 [19]. The results of both TTE and CMR were interpreted by specialists in cardiac imaging or genetic cardiomyopathies, blinded to the thyroid status of patients.

### Follow-up and outcomes

Follow-up data were collected by trained clinical staff via telephone interviews and/or medical records of outpatient visits. The primary outcome was a composite of cardiovascular mortality and heart transplantation. This study also evaluated two secondary outcomes: all-cause mortality and major adverse cardiovascular events (MACE). MACE was defined as a combination of the following clinical endpoints: (1) cardiovascular mortality; (2) heart transplantation; (3) cardiac resynchronization therapy (CRT) implantation; (4) malignant arrhythmia: aborted SCD, ventricular fibrillation (VF), sustained ventricular tachycardia (VT), or appropriate implantable cardioverter-defibrillator (ICD) therapy; and (5) thromboembolism: embolic stroke, transient ischemic attack, embolic myocardial infarction, or peripheral artery embolism. Outcomes were sought systematically from pertinent medical records or death certificates by trained clinical staff.

### Statistical analysis

For descriptive analysis, continuous variables were expressed as mean ± standard deviation or median [25th–75th percentile], and compared using the independent sample t-test or the Mann–Whitney U test. Categorical variables were presented as proportions, and compared using the chi-square test or the Fisher’s exact test. Follow-up duration was calculated from the date of LVNC diagnosis to the date of censoring or reaching clinical endpoints. Univariable and multivariable Cox proportional hazards regression models were used to estimate hazard ratios of adverse outcomes for clinical variables. We further applied restricted cubic spline (RCS) models fitted for aforementioned Cox proportional hazards models to explore the potential nonlinear association between FT3 levels and adverse outcomes. The LVNC cohort was stratified into three groups according to tertiles of serum FT3, and the cumulative incidence of both primary and secondary outcomes in the three groups was evaluated using the Kaplan–Meier curves. In the multivariable analyses to predict adverse outcomes in LVNC according to tertiles of FT3 levels, Model 1 adjusted for age and gender; Model 2 adjusted for age, gender, cardiovascular risk factors, cardiac thrombosis and non-sustained VT (NSVT); and Model 3 further adjusted for LAD, LVEDD, and LVEF in addition to covariates in Model 2. Subgroup analysis was performed for age, gender, occurrence of atrial fibrillation (AF) or NSVT, LAD, LVEDD, and LVEF to predict the risk of primary outcome in LVNC according to tertiles of serum FT3. In order to determine if FT3 provided additional predictive value for MACE when incorporated into a pre-existing risk score [21] for LVNC, the area under the curve (AUC), the absolute integrated discrimination improvement (IDI), and the net reclassification index (NRI) were calculated to assess the enhancement in accuracy and discrimination. All analyses were conducted using SAS V.9.4 (SAS Institute, Cary, NC, USA) and RStudio V.1.1.414 (Boston, MA, USA). Statistical significance was defined by a two-tailed *P*-value < 0.05.

## Results

### Baseline characteristics

The final cohort was comprised of 388 individuals with complete thyroid function profiles who met imaging diagnostic criteria for LVNC (Fig. 1). No significant differences were noted between the included (N = 388) and excluded (N = 48) patients in terms of demographics, comorbidity, cardiac function, and major TTE results (Supplemental Table S1). All participants underwent TTE evaluation, and CMR was performed in 327 patients (84.3%). Baseline characteristics of patients according to the occurrence of the primary outcome were summarized in Table 1. The mean age at diagnosis was 43.5 ± 17.0 years, with 253 patients (65.2%) being male. Around 60% of the population had at least one risk factor for cardiovascular diseases (hypertension, dyslipidemia, diabetes mellitus, or smoking). Patients typically experienced dyspnea, palpitation and edema as their primary clinical manifestations. A number of individuals presented with dilated left chambers and decreased LVEF, and approximately 10% of the participants were diagnosed with cardiac thrombosis. The apex and the lateral wall of left ventricle were the most frequently affected regions of noncompaction.

**Table 1:** Baseline characteristics of LVNC cohort according to the occurrence of primary outcome

	Overall (N = 388)	Primary outcome (cardiovascular mortality and/or heart transplantation)		
		Positive (N = 98)	Negative (N = 290)	*P*-value
Demographics				
Age (years)	43.5 ± 17.0	46.8 ± 17.4	42.4 ± 16.7	0.024
Male, *n* (%)	253 (65.2%)	72 (73.5%)	181 (62.4%)	0.047
Body mass index (kg/m^2^)	23.37 ± 4.13	22.58 ± 4.19	23.64 ± 4.09	0.030
Cardiovascular risk factors				
Hypertension, *n* (%)	105 (27.1%)	19 (19.4%)	86 (29.7%)	0.048
Hyperlipidemia,* n* (%)	116 (29.9%)	31 (31.6%)	85 (29.3%)	0.664
Diabetes Mellitus, *n* (%)	61 (15.7%)	22 (22.4%)	39 (13.4%)	0.034
Smoking, *n* (%)	124 (32.0%)	40 (40.8%)	84 (29.0%)	0.030
Cardiovascular risk factors*, *n* (%)	232 (59.8%)	62 (63.3%)	170 (58.6%)	0.418
Clinical features				
Dyspnea, *n* (%)	295 (76.0%)	87 (88.8%)	208 (71.7%)	0.001
Chest Pain, *n* (%)	61 (15.7%)	24 (24.5%)	37 (12.8%)	0.006
Palpitation, *n* (%)	199 (51.3%)	41 (41.8%)	158 (54.5%)	0.030
Syncope,* n* (%)	46 (11.9%)	11 (11.2%)	35 (12.1%)	0.823
Edema,* n* (%)	80 (20.6%)	29 (29.6%)	51 (17.6%)	0.011
NYHA class III/IV, *n* (%)	206 (53.1%)	78 (79.6%)	128 (44.1%)	< 0.001
Medical Treatment				
Beta-Blockers,* n* (%)	317 (81.7%)	83 (84.7%)	234 (80.7%)	0.375
Calcium channel blockers,* n* (%)	21 (5.4%)	9 (9.2%)	12 (4.1%)	0.056
ARNI/ACEI/ARB, *n* (%)	263 (67.8%)	62 (63.3%)	201 (69.3%)	0.268
Diuretics,* n* (%)	282 (72.7%)	90 (91.8%)	192 (66.2%)	< 0.001
MRA,* n* (%)	272 (70.1%)	83 (84.7%)	189 (65.2%)	< 0.001
Oral Anticoagulants, *n* (%)	88 (22.7%)	24 (24.5%)	64 (22.1%)	0.621
Laboratory test				
FT3 (pg/mL)	2.86 ± 0.53	2.60 ± 0.55	2.95 ± 0.49	< 0.001
TT3 (ng/mL)	1.00 ± 0.26	0.90 ± 0.29	1.02 ± 0.23	< 0.001
FT4 (ng/dL)	1.21 ± 0.20	1.25 ± 0.23	1.20 ± 0.19	0.037
TT4 (ug/dL)	7.72 ± 1.77	7.77 ± 2.07	7.71 ± 1.66	0.801
TSH (mIU/L)	1.97 (1.12–3.39)	1.70 (0.98–3.09)	2.10 (1.23–3.43)	0.033
NT-pro BNP (pg/mL)	1090.8 (246.7–2793.8)	3057.0 (1668.8–4943.2)	630.0 (134.7–1636.9)	< 0.001
Albumin (g/L)	43.7 (40.1–46.5)	41.9 (38.1–44.7)	44.1 (41.1–46.7)	< 0.001
Alanine Aminotransferase (IU/L)	22.0 (15.5–37.0)	26.0 (17.0–40.0)	21.0 (14.0–34.3)	0.045
Creatinine (μmol/L)	82.2 (67.7–98.0)	84.6 (70.7–101.6)	81.0 (66.3–97.3)	0.139
Glucose (mmol/L)	5.04 (4.58–5.68)	5.01 (4.52–5.86)	5.04 (4.58–5.58)	0.654
ECG				
QRS Duration (ms)	114.5 ± 31.3	119.2 ± 32.3	113.0 ± 30.9	0.098
QT_C_ (ms)	451.0 ± 47.5	456.3 ± 46.8	449.3 ± 47.7	0.216
AFL/AF, *n* (%)	72 (18.6%)	20 (20.4%)	52 (17.9%)	0.586
NSVT, *n* (%)	101 (26.0%)	33 (33.7%)	68 (23.4%)	0.046
LBBB, *n* (%)	48 (12.4%)	13 (13.3%)	35 (12.1%)	0.756
III°AVB, *n* (%)	12 (3.1%)	5 (5.1%)	7 (2.4%)	0.184
TTE				
LAD (mm)	41.3 ± 8.4	45.9 ± 7.7	39.8 ± 8.1	< 0.001
LVEDD (mm)	61.1 ± 10.5	66.1 ± 10.2	59.4 ± 10.1	< 0.001
LVEF (%)	40.6 ± 14.6	32.0 ± 10.6	43.5 ± 14.7	< 0.001
Cardiac thrombosis, *n* (%)	40 (10.3%)	16 (16.3%)	24 (8.3%)	0.023
CMR (N = 327)				
LAD (mm)	36.7 ± 10.5	43.1 ± 9.9	34.9 ± 9.9	< 0.001
LVEDD (mm)	64.8 ± 11.1	71.5 ± 9.2	62.9 ± 10.9	< 0.001
LVEF (%)	32.4 ± 14.5	23.4 ± 9.5	35.1 ± 14.6	< 0.001
LVEDV (mL)	232.0 ± 106.1	289.5 ± 98.2	214.6 ± 102.4	< 0.001
LVESV (mL)	165.6 ± 99.9	225.1 ± 89.8	147.6 ± 95.9	< 0.001
CO (L/minute)	4.70 ± 2.01	4.74 ± 2.08	4.69 ± 1.99	0.876
LGE (+), *n* (%)	188 (57.5%)	55 (56.1%)	133 (45.9%)	0.006
Extent of noncompaction				
Apical noncompaction, *n* (%)	302 (77.8%)	76 (77.6%)	226 (77.9%)	0.938
LVAW Noncompaction, *n* (%)	65 (16.8%)	18 (18.4%)	47 (16.2%)	0.621
LVIW noncompaction, *n* (%)	63 (16.2%)	16 (16.3%)	47 (16.2%)	0.978
LVLW noncompaction, *n* (%)	325 (83.8%)	84 (85.7%)	241 (83.1%)	0.545
LVPW noncompaction, *n* (%)	45 (11.6%)	16 (16.3%)	29 (10.9%)	0.091
Septal noncompaction, *n* (%)	12 (3.1%)	1 (1.0%)	11 (3.8%)	0.309

LVNC, left ventricular noncompaction; NYHA, New York Heart Association; ARNI, angiotensin receptor-neprilysin inhibition; ACEI, angiotensin-converting enzyme inhibitor; ARB, angiotensin receptor blocker; MRA, mineralocorticoid receptor antagonist; FT3, free triiodothyronine; TT3, total triiodothyronine; FT4, free thyroxine; TT4, total thyroxine; TSH, thyrotropin; NT-pro BNP, N-terminal pro-brain natriuretic peptide; ECG, electrocardiogram; QTc, corrected QT; AFL, atrial flutter; AF, atrial fibrillation; NSVT, nonsustained ventricular tachycardia; LBBB, left bundle branch block; AVB, atrioventricular block; TTE, transthoracic echocardiography; LAD, left atrial diameter; LVEDD, left ventricular end-diastolic diameter; LVEF, left ventricular ejection fraction; CMR, cardiac magnetic resonance; LVEDV, left ventricular end-diastolic volume; LVESV, left ventricular end-systolic volume; CO, cardiac output; LGE (+), positive late gadolinium enhancement; LVAW, left ventricular anterior wall; LVIW, left ventricular inferior wall; LVLW, left ventricular lateral wall; and LVPW, left ventricular posterior wall. Cardiovascular risk factors were defined as a composite of hypertension, dyslipidemia, diabetes mellitus, or smoking

### Primary and secondary outcomes

Over a median follow-up of 5.22 years (interquartile range: 3.56–7.35 years), the primary outcome occurred in 98 (25.3%) patients, with the observational rates of cardiovascular mortality and heart transplantation being 18.6% and 7.2%, respectively. As for secondary outcomes, 130 (33.5%) patients presented with at least one MACE event, and 75 (19.3%) patients had died at last follow-up. As was shown in Supplemental Table S2, 5.4% of individuals were implanted with CRT due to heart failure. Sustained VT (n = 23, 5.9%) was the most frequent malignant arrhythmia, followed by aborted SCD/VF (n = 15, 3.9%) and appropriate ICD therapy (n = 12, 3.1%). Nine participants (2.3%) experienced the onset of new thromboembolic events.

### Prognostic variables for adverse outcomes in LVNC

According to Table 1, LVNC patients who experienced adverse outcomes of cardiovascular death and/or heart transplantation were relatively older with lower body mass index (BMI), suffered from more severe symptoms of cardiac dysfunction, and displayed enlarged left atrium and ventricle, reduced LVEF, and higher proportions of NSVT episodes and cardiac thrombosis. For the laboratory data, decreased levels of FT3, TSH, and albumin, as well as elevated circulating N-terminal pro-brain natriuretic peptide (NT-pro BNP) and alanine aminotransferase (ALT) were observed in patients with primary endpoints. Tables 2 and 3 illustrated associations between the risk of adverse outcomes and potential clinical factors. Variables with medical relevance or statistical significance (*P* < 0.05) in relation to clinical outcomes were included in multivariable Cox regression analyses. Serum FT3 levels at baseline, LAD and LVEF on TTE were demonstrated to be independently associated with the risk for both primary and secondary outcomes in our study (Tables 2 and 3). Data of NT-pro BNP were available for 346 (89.2%) participants in our cohort (Supplemental Table S3). After incorporating NT-pro BNP into the multivariable model, serum FT3 levels at baseline still remained an independent factor for adverse outcomes in LVNC (Supplemental Table S4 and S5).

**Table 2:** Cox regression analysis on risk factors for the primary outcome in LVNC

Variables	Primary outcome (cardiovascular mortality and/or heart Transplantation)					
	Univariable analysis			Multivariable analysis		
	HR	95% CI	*P-*value	HR	95% CI	*P-*value
Age^a^	1.013	1.001–1.026	0.033			
Male^a^	1.496	0.955–2.343	0.079			
Cardiovascular risk factors*	1.188	0.787–1.792	0.413			
NYHA III or IV^a^	3.694	2.258–6.042	< 0.001			
FT3^a^	0.379	0.272–0.529	< 0.001	0.455	0.313–0.664	< 0.001
FT4^a^	3.213	1.194–8.648	0.021			
Ln TSH^a^	0.777	0.621–0.973	0.028			
AFL/AF	1.192	0.729–1.948	0.485			
NSVT^a^	1.570	1.032–2.390	0.035			
LAD (TTE)^a^	1.069	1.047–1.091	< 0.001	1.052	1.027–1.078	< 0.001
LVEDD (TTE)^a^	1.046	1.029–1.063	< 0.001			
LVEF (TTE)^a^	0.949	0.934–0.965	< 0.001	0.964	0.947–0.981	< 0.001
Cardiac thrombosis^a^	2.039	1.192–3.488	0.009			

LVNC, left ventricular noncompaction; HR, hazard ratio; CI, confidence interval; NYHA, New York Heart Association; FT3, free triiodothyronine; FT4, free thyroxine; TSH, thyrotropin; AFL, atrial flutter; AF, atrial fibrillation; NSVT, nonsustained ventricular tachycardia; TTE, transthoracic echocardiography; LAD, left atrial diameter; LVEDD, left ventricular end-diastolic diameter; and LVEF, left ventricular ejection fraction. * Cardiovascular risk factors were defined as a composite of hypertension, dyslipidemia, diabetes mellitus, or smoking. ^a^Variables included in multivariable analysis on the primary outcome

**Table 3:** Cox regression analysis on risk factors for secondary outcomes in LVNC

Variables	All-cause mortality						MACE					
	Univariable analysis			Multivariable analysis			Univariable analysis			Multivariable analysis		
	HR	95% CI	*P-*value	HR	95% CI	*P-*value	HR	95% CI	*P-*value	HR	95% CI	*P-*value
Age^a,b^	1.039	1.023–1.055	< 0.001	1.034	1.017–1.051	< 0.001	1.018	1.007–1.029	0.001			
Male^a,b^	1.677	0.987–2.850	0.056				1.099	0.759–1.593	0.616			
Cardiovascular risk factors*^,a^	1.743	1.059–2.869	0.029				1.243	0.869–1.779	0.234			
NYHA III or IV^a,b^	2.987	1.737–5.136	< 0.001				3.066	2.046–4.594	< 0.001			
FT3^a,b^	0.407	0.277–0.597	< 0.001	0.547	0.349–0.858	0.009	0.518	0.381–0.704	< 0.001	0.663	0.475–0.925	0.016
FT4^b^	1.928	0.630–5.895	0.250				2.789	1.185–6.562	0.019			
Ln TSH^a^	0.722	0.559–0.932	0.012				0.902	0.741–1.098	0.305			
AFL/AF	1.410	0.820–2.422	0.214				1.364	0.902–2.063	0.141			
NSVT^a,b^	2.210	1.389–3.516	0.001				1.593	1.105–2.298	0.013			
LAD (TTE)^a,b^	1.054	1.029–1.080	< 0.001	1.039	1.008–1.070	0.012	1.054	1.035–1.073	< 0.001	1.036	1.015–1.059	0.001
LVEDD (TTE)^a,b^	1.037	1.018–1.057	< 0.001				1.039	1.024–1.055	< 0.001			
LVEF (TTE)^a,b^	0.958	0.941–0.975	< 0.001	0.971	0.951–0.991	0.004	0.957	0.944–0.970	< 0.001	0.966	0.952–0.980	< 0.001
Cardiac Thrombosis^b^	0.897	0.412–1.953	0.784				1.740	1.068–2.834	0.026			

LVNC, left ventricular noncompaction; MACE, major adverse cardiovascular events; HR, hazard ratio; CI, confidence interval; NYHA, New York Heart Association; FT3, free triiodothyronine; FT4, free thyroxine; TSH, thyrotropin; AFL, atrial flutter; AF, atrial fibrillation; NSVT, nonsustained ventricular tachycardia; TTE, transthoracic echocardiography; LAD, left atrial diameter; LVEDD, left ventricular end-diastolic diameter; and LVEF, left ventricular ejection fraction. * Cardiovascular risk factors were defined as a composite of hypertension, dyslipidemia, diabetes mellitus, or smoking. ^a^Variables included in the multivariable analysis on all-cause mortality. ^b^Variables included in the multivariable analysis on MACE

### Dose–response association between FT3 levels and adverse outcomes in LVNC

**Fig. 2:**
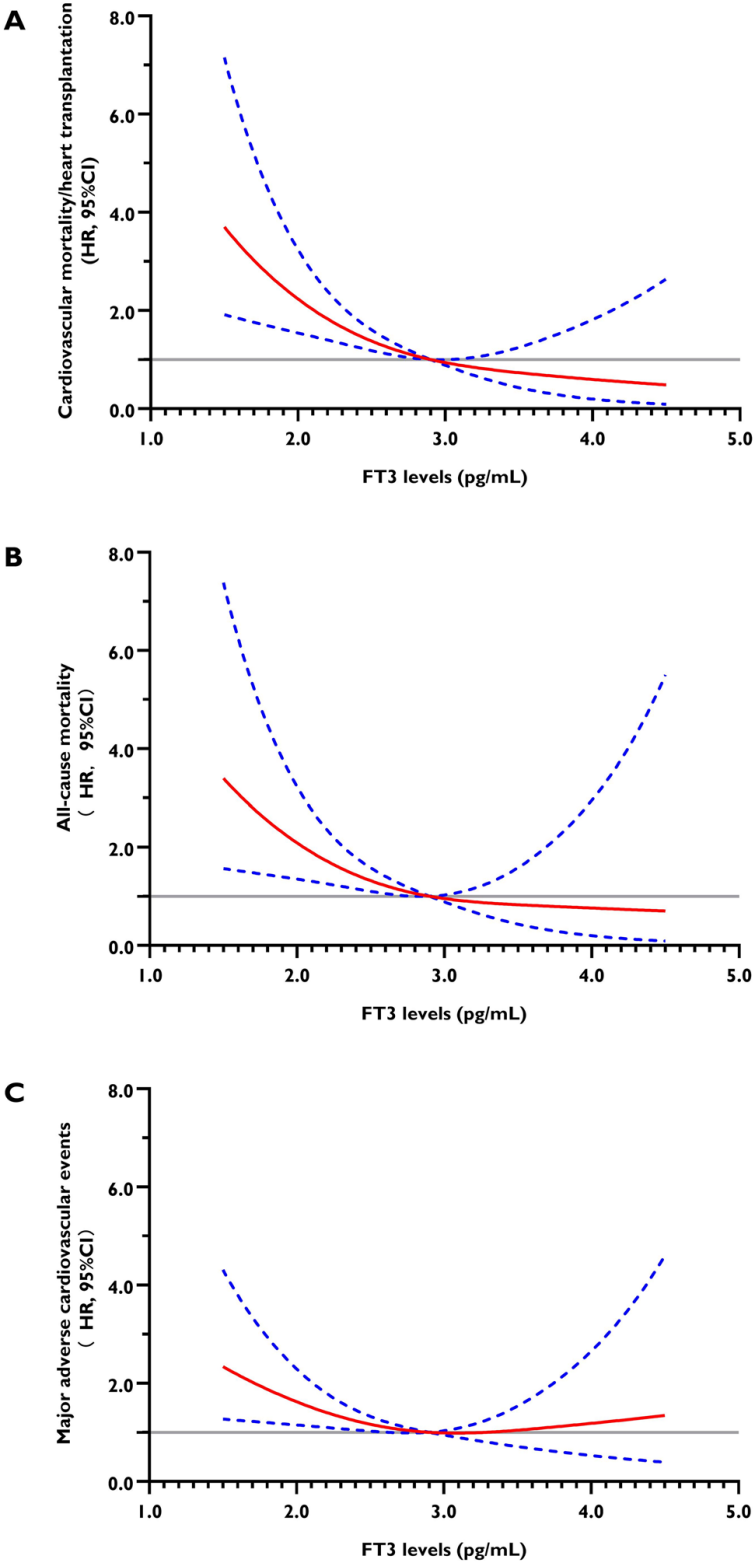
Adjusted dose–response associations between FT3 levels and hazard ratios for the primary and secondary outcomes of the LVNC cohort: (**A**) cardiovascular mortality and/or heart transplantation, (**B**) all-cause mortality, and (**C**) major adverse cardiovascular events. LVNC, left ventricular noncompaction; FT3, free triiodothyronine; HR, hazard ratios; and CI, confidence interval

RCS regression analysis indicated an L-shaped relationship between FT3 levels and hazard ratios for cardiovascular mortality/heart transplantation and all-cause mortality, and a U-shaped relationship between FT3 levels and hazard ratios for MACE. As was depicted by Fig. 2, lower FT3 levels significantly increased the risk for both primary and secondary outcomes in patients with LVNC.

### Clinical features and outcomes of LVNC patients stratified by tertiles of FT3 levels

**Fig. 3:**
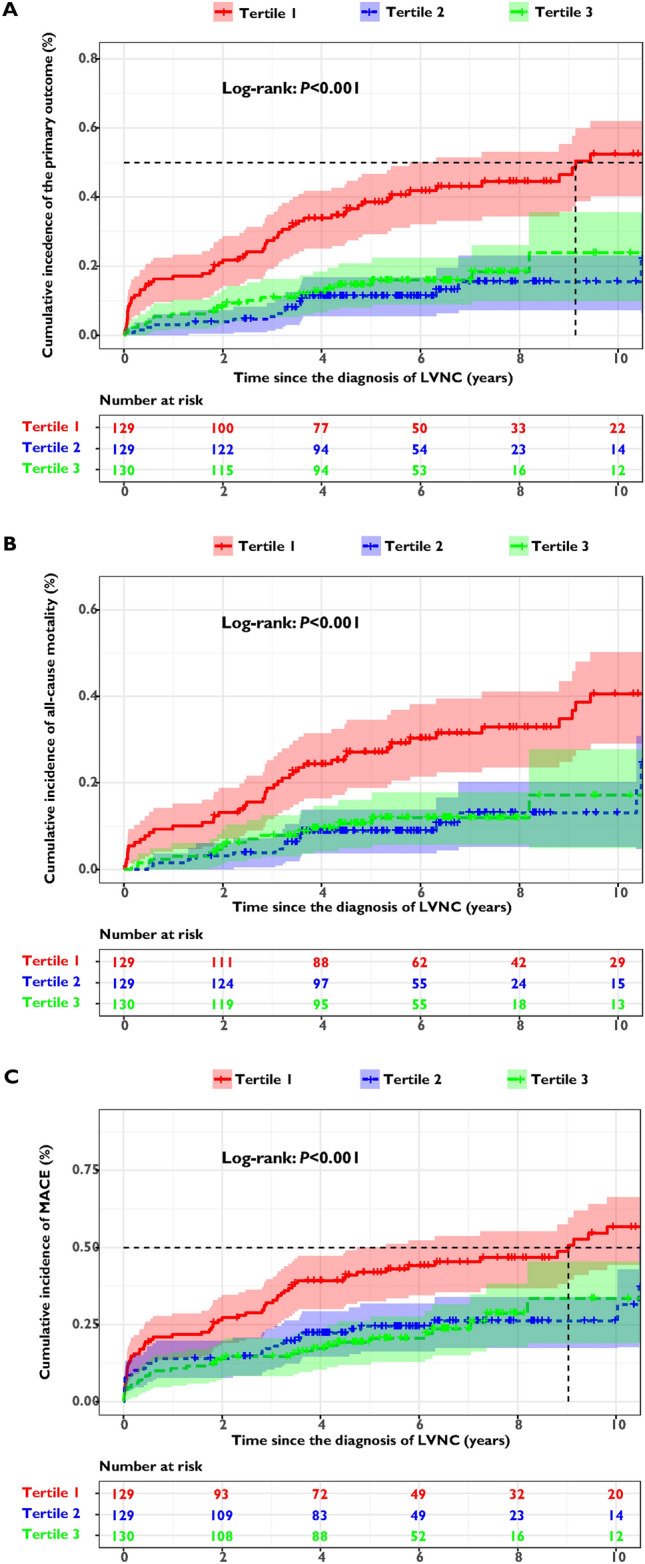
Kaplan–Meier curves illustrating the cumulative incidence of the primary and secondary outcomes in the LVNC cohort according to tertiles of FT3 levels: (**A**) cardiovascular mortality and/or heart transplantation, (**B**) all-cause mortality, and (**C**) major adverse cardiovascular events. LVNC, left ventricular noncompaction; FT3, free triiodothyronine; and MACE, major adverse cardiovascular events. Primary outcome: cardiovascular mortality and/or heart transplantation. Tertile 1: FT3 < 2.69 pg/mL; Tertile 2: FT3 2.69–3.09 pg/mL; Tertile 3: FT3 > 3.09 pg/mL

**Fig. 4:**
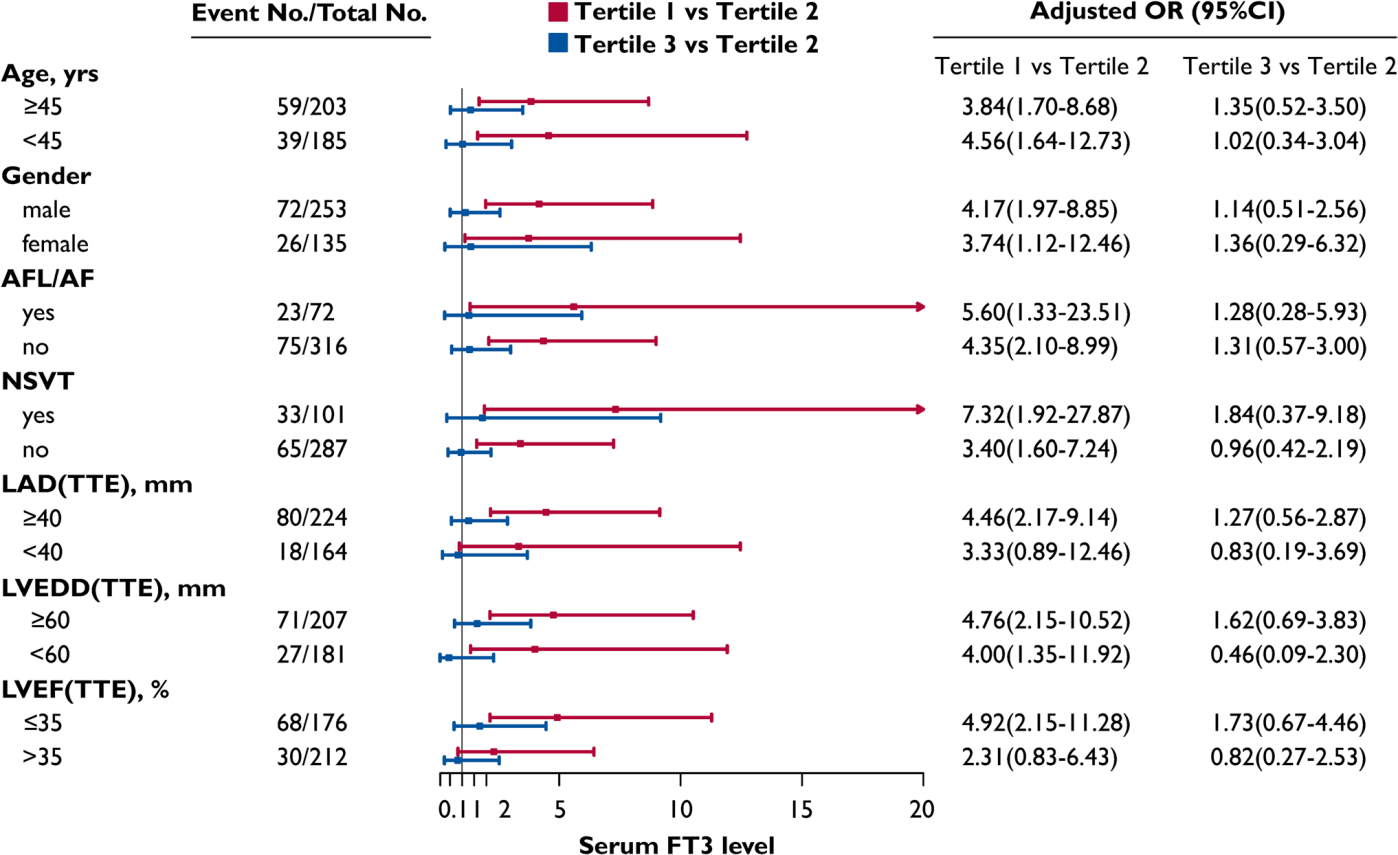
Subgroup analysis on cardiovascular mortality and/or heart transplantation of the LVNC cohort according to tertiles of FT3 levels. LVNC, left ventricular noncompaction; FT3, free triiodothyronine; OR, odds ratio, CI, confidence interval; AFL, atrial flutter; AF, atrial fibrillation; NSVT, nonsustained ventricular tachycardia; TTE, transthoracic echocardiography; LAD, left atrial diameter; LVEDD, left ventricular end-diastolic diameter; and LVEF, left ventricular ejection fraction. Tertile 1: FT3 < 2.69 pg/mL; Tertile 2: FT3 2.69–3.09 pg/mL; Tertile 3: FT3 > 3.09 pg/mL

The LVNC cohort was divided into three groups according to tertiles of FT3 levels: Tertile 1 (T1), those with lower FT3 levels (FT3 < 2.69 pg/mL, N = 129); Tertile 2 (T2), those with normal FT3 levels (FT3 2.69–3.09 pg/mL, N = 129); and Tertile 3 (T3), those with higher FT3 levels (FT3 > 3.09 pg/mL, N = 130). As was shown in Table 4, there were no significant differences in age, gender, and cardiovascular risk factors between the T1 and T2 group. Compared with the T2 group (FT3 levels were all within normal reference ranges), patients with lower FT3 levels in the T1 group displayed enlarged left atrium and ventricle, significantly reduced LVEF, and elevated proportion of NYHA class III or IV. Kaplan–Meier curves revealed that the cumulative incidence of adverse outcomes in the T1 group was significantly higher than the T2 and T3 groups (Log rank *P* < 0.001) (Fig. 3). As was illustrated in Table 5, compared with patients in the T2 group, the crude odds ratios (ORs) of the T1 group were 4.315 (95% CI: 2.392–7.783, *P* < 0.001), 3.294 (95% CI: 1.758–6.172, *P* < 0.001) and 2.544 (95% CI: 1.517–4.265, *P* < 0.001) for the primary and secondary outcomes. After adjusting for potential confounders, the adjusted ORs of the T1 group were 3.822 (95% CI: 2.003–7.296, *P* < 0.001) for cardiovascular mortality/heart transplantation, 2.622 (95% CI: 1.326–5.185, *P* = 0.006) for all-cause mortality, and 1.997 (95% CI: 1.132–3.523,* P* = 0.017) for the occurrence of MACE. Subgroup analysis demonstrated that, for patients with LAD ≥ 40 mm or LVEF ≤ 35%, the risk of primary outcome in the T1 group was significantly higher than the T2 group, with the adjusted ORs of 4.458 (95% CI: 2.174–9.141, *P* < 0.001) and 4.920 (95% CI: 2.146–11.277, *P* < 0.001), respectively (Fig. 4).

**Table 4:** Clinical features and outcomes of LVNC cohort stratified by tertiles of FT3 levels

	Overall (N = 388)	Tertile 1 (N = 129)	Tertile 2 (N = 129)	Tertile 3 (N = 130)	*P-*value
Clinical parameters					
Age (years)	43.5 ± 17.0	47.4 ± 16.9	45.5 ± 15.4	37.6 ± 17.1	A, b, c
Male,* n* (%)	253 (65.2%)	78 (60.5%)	76 (58.9%)	99 (76.2%)	A, b, c
Cardiovascular Risk Factors *,* n* (%)	232 (59.8%)	85 (65.9%)	75 (58.1%)	72 (55.4%)	NS
NYHA III or IV,* n* (%)	206 (53.1%)	92 (71.3%)	60 (46.5%)	54 (41.5%)	A, a, b
QTc (ms)	451.0 ± 47.5	456.0 ± 47.0	451.3 ± 53.7	445.6 ± 40.9	NS
AFL/AF,* n* (%)	72 (18.6%)	23 (17.8%)	22 (17.1%)	27 (20.8%)	NS
NSVT,* n* (%)	101 (26.0%)	42 (32.6%)	29 (22.5%)	30 (23.1%)	NS
LAD on TTE (mm)	41.3 ± 8.4	43.3 ± 7.8	41.1 ± 8.4	39.6 ± 8.7	A, b
LVEDD on TTE (mm)	61.1 ± 10.5	62.3 ± 11.0	60.3 ± 10.4	60.6 ± 10.1	NS
LVEF on TTE (%)	40.6 ± 14.6	35.6 ± 13.2	42.6 ± 15.0	43.6 ± 14.5	A, a, b
Cardiac thrombosis,* n* (%)	40 (10.3%)	16 (12.4%)	12 (9.3%)	12 (9.2%)	NS
LGE,* n* (%) (N = 327)	188 (57.5%)	66 (62.9%)	60 (55.6%)	62 (54.4%)	NS
Clinical outcomes					
Primary outcome (composite),* n* (%)	98 (25.3%)	57 (44.2%)	20 (15.5%)	21 (16.2%)	A, a, b
Secondary outcome					
All-cause mortality,* n* (%)	75 (19.3%)	43 (33.3%)	17 (13.2%)	15 (11.5%)	A, a, b
MACE,* n* (%)	130 (33.5%)	64 (49.6%)	36 (27.9%)	30 (23.1%)	A, a, b
Cardiovascular mortality,* n* (%)	72 (18.6%)	42 (43.6%)	15 (11.6%)	15 (11.5%)	A, a, b
Heart transplantation,* n* (%)	28 (7.2%)	16 (12.4%)	5 (3.9%)	7 (5.4%)	A, a, b
CRT implantation,* n* (%)	21 (5.4%)	6 (4.7%)	11 (8.5%)	4 (3.1%)	NS
Aborted SCD/VF,* n* (%)	15 (3.9%)	10 (7.8%)	2 (1.6%)	3 (2.3%)	A
Sustained VT,* n* (%)	23 (5.9%)	10 (7.8%)	8 (6.2%)	5 (3.8%)	NS
Appropriate ICD therapy,* n* (%)	12 (3.1%)	3 (2.3%)	6 (4.7%)	3 (2.3%)	NS
Thromoembolism,* n* (%)	9 (2.3%)	3 (2.3%)	3 (2.3%)	3 (2.3%)	NS

LVNC, left ventricular noncompaction; FT3, free triiodothyronine; NYHA, New York Heart Association; QTc, corrected QT; AFL, atrial flutter; AF, atrial fibrillation; NSVT, nonsustained ventricular tachycardia; TTE, transthoracic echocardiography; LAD, left atrial diameter; LVEDD, left ventricular end-diastolic diameter; LVEF, left ventricular ejection fraction; LGE (+), positive late gadolinium enhancement; MACE, major adverse cardiovascular events; CRT, cardiac resynchronization therapy; SCD, sudden cardiac death; VF, ventricular fibrillation; VT, ventricular tachycardia; and ICD, implantable cardioverter defibrillator. *Cardiovascular risk factors were defined as a composite of hypertension, dyslipidemia, diabetes mellitus, or smoking. Tertile 1: FT3 < 2.69 pg/mL; Tertile 2: FT3 2.69–3.09 pg/mL; Tertile 3:FT3 > 3.09 pg/mL. Primary outcome: cardiovascular mortality and/or heart transplantation. For *P*-values: A, significant difference between the three groups; a, significant difference between the tertile 1 group and the tertile 2 group; b, significant difference between the tertile 1 group and the tertile 3 group; c, significant difference between the tertile 2 group and the tertile 3 group; NS, not significant

**Table 5:** Multivariable analysis to predict adverse clinical outcomes of the LVNC cohort according to tertiles of FT3 levels

Clinical outcomes	Events N (%)	Unadjusted		Model 1		Model 2		Model 3	
		OR (95%CI)	*P-*value	OR (95%CI)	*P-*value	OR (95%CI)	*P-*value	OR (95%CI)	*P-*value
Cardiovascular mortality and/or heart transplantation									
Tertile 1	57/129 (45.7)	4.315 (2.392–7.783)	< 0.001	4.343 (2.388–7.899)	< 0.001	4.238 (2.317–7.751)	< 0.001	3.822 (2.003–7.296)	< 0.001
Tertile 2	20/129 (15.5)	Reference		Reference		Reference		Reference	
Tertile 3	21/130 (16.2)	1.050 (0.539–2.047)	0.886	1.020 (0.514–2.028)	0.954	1.019 (0.510–2.035)	0.957	1.259 (0.606–2.615)	0.537
All-cause mortality									
Tertile 1	43/129 (33.3)	3.294 (1.758–6.172)	< 0.001	3.247 (1.692–6.232)	< 0.001	3.104 (1.610–5.985)	0.001	2.622 (1.326–5.185)	0.006
Tertile 2	17/129 (13.2)	Reference		Reference		Reference		Reference	
Tertile 3	15/130 (11.5)	0.859 (0.409–1.804)	0.689	1.003 (0.464–2.169)	0.994	0.966 (0.445–2.094)	0.929	1.080 (0.485–2.404)	0.851
MACE									
Tertile 1	64/129 (49.6)	2.544 (1.517–4.265)	< 0.001	2.497 (1.479–4.213)	0.001	2.416 (1.424–4.100)	0.001	1.997 (1.132–3.523)	0.017
Tertile 2	36/129 (27.9)	Reference		Reference		Reference		Reference	
Tertile 3	30/130 (23.1)	0.775 (0.442–1.358)	0.373	0.835 (0.468–1.490)	0.541	0.832 (0.464–1.493)	0.538	0.960 (0.515–1.790)	0.898

LVNC, left ventricular noncompaction; FT3, free triiodothyronine; MACE, major adverse cardiovascular events; OR, odds ratio, and CI, confidence interval. Tertile 1: FT3 < 2.69 pg/mL; Tertile 2: FT3 2.69–3.09 pg/mL; Tertile 3: FT3 > 3.09 pg/mL. Model 1 adjusted for age and gender. Model 2 adjusted for age, gender, cardiovascular risk factors, cardiac thrombosis and nonsustained ventricular tachycardia. Model 3 adjusted for age, gender, cardiovascular risk factors, cardiac thrombosis, nonsustained ventricular tachycardia, left atrial diameter, left ventricular end-diastolic diameter, and left ventricular ejection fraction. Cardiovascular risk factors were defined as a composite of hypertension, dyslipidemia, diabetes mellitus, or smoking

### Predictive value of FT3 for MACE in LVNC

Incorporating FT3 into a pre-existing risk score for MACE [21] in LVNC further improved its predictive performance. As was shown in Table 6, analysis by receiver-operating characteristic curve confirmed the predictive ability of the previously established risk score (AUC 0.691, 95% CI: 0.637–0.744, *P* < 0.001). When adding FT3 to this published risk score [21], the combined model displayed enhanced capability in both accuracy and discrimination (AUC 0.713, 95% CI: 0.661–0.766, *P* < 0.001; IDI 0.026, 95% CI: 0.009–0.043, *P* = 0.002; NRI 0.217, 95% CI: 0.007–0.426,* P* = 0.044).

**Table 6:** Model improvement for the published MACE prediction score of LVNC in combination with FT3

Risk prediction scores	AUC		IDI		NRI	
	Estimate (95%CI)	*P-*value	Estimate (95%CI)	*P-*value	Estimate (95%CI)	*P-*value
Published Score^21^	0.691 (0.637–0.744)	< 0.001	Reference		Reference	
Published Score^21^ + FT3	0.713 (0.661–0.766)	< 0.001	0.026 (0.009–0.043)	0.002	0.217 (0.007–0.426)	0.044

LVNC, left ventricular noncompaction; MACE, major adverse cardiovascular events; FT3, free triiodothyronine; AUC, area under the curve; IDI, integrated discrimination improvement; NRI, net reclassification index; and CI, confidence interval

## Discussion

In the current study, we explored the prognostic value of THs in a large cohort of morphologically diagnosed LVNC patients, and identified that low FT3 level at baseline was significantly associated with adverse cardiovascular outcomes. Lower FT3 status (FT3 < 2.69 pg/mL) was not only linked to more severe cardiac dysfunction and remodeling, but also strongly related to higher incidence of mortality and MACE. Adding FT3 to the previously reported risk score for MACE [21] in LVNC further improved its predictive performance.

THs play a crucial role in maintaining cardiovascular homeostasis from prenatal period to death. In critically ill patients, low serum T3 levels with normal or slightly decreased TSH levels are commonly observed, which is called “low T3 syndrome”, or “nonthyroidal illness syndrome”. This thyroid status is due to a complex adaptive mechanism of the hypothalamic-pituitary-thyroid (HPT) axis to a wide array of severe clinical conditions, and the extent of decrease in T3 generally represent the severity of the disease [22–24]. Low T3 syndrome has been extensively described in the context of several cardiovascular diseases such as heart failure [25], acute myocardial infarction [26], ischemic heart disease [27], and acute myocarditis [28]. In the current study, we found that LVNC patients with worse outcomes also displayed lower levels of serum FT3 and TSH. Patients who experienced adverse cardiovascular events in our cohort presented with decreased BMI and serum albumin, elevated concentrations of ALT and creatinine, remarkably enlarged left heart chambers, significantly reduced LVEF, and deterioration of NYHA functional class. The severe clinical condition in these patients can cause a downregulation of the HPT axis at both the hypothalamic and pituitary levels, and the conversion of FT4 into FT3 through 5’-monodeiodinase in peripheral tissues is usually reduced [29, 30], eventually contributing to the decline in serum FT3 concentrations. Moreover, since type I 5’-monodeiodinase is especially enriched in the liver and kidneys [31], the impairment of liver and kidney function due to cardiac failure in LVNC patients with worse outcomes may further aggravate their low T3 status.

On the other hand, low serum FT3 level has been identified as a strong predictor for adverse outcomes in numerous cardiovascular diseases, including both acute decompensated and chronic stable heart failure [5–7], dilated and hypertrophic cardiomyopathies [8, 9], as well as acute coronary syndrome [11]. In the current study, we demonstrated for the first time that low FT3 levels at baseline could provide prognostic information for the risk of long-term mortality and MACE in patients with LVNC. Underlying mechanisms were far from elucidated, but our findings indicated that FT3 might be associated with the development of cardiovascular complications in LVNC.

First, FT3 may be involved in the myocyte remodeling and cardiac failure in LVNC. Earlier studies have revealed that THs not only possess the ability to promote the beneficial shaping of cardiomyocyte [32], but also can improve the cardiac systolic function via genomic and nongenomic approaches [1]. Decreased FT3 is responsible for cardiomyocyte lengthening, impaired myocardial contractility, declined cardiac output, and worsened hemodynamic status [2]. As was shown in our study, LVNC patients with lower FT3 levels in the T1 group presented with severe cardiac dysfunction and remodeling, and encountered more adverse events related to heart failure.

Second, FT3 may be correlated with myocardial fibrosis and ventricular arrhythmia in LVNC. Myocardial fibrosis is a crucial arrhythmogenic substrate of life-threatening cardiac arrhythmias. Previous research has found that THs tend to inhibit or reverse myocardial fibrosis by stimulating the breakdown of interstitial collagen through the activity of matrix metalloproteinases [33]. In hypothyroidism, elevated accumulation of collagen has been observed due to the up-regulation of pro-α1 collagen gene expression [34]. Meanwhile, the QT interval is often prolonged because of the lengthened ventricular action potential [35], causing increased susceptibility to ventricular arrhythmia. Data from FT3 stratified groups of our study illustrated that, as serum FT3 levels declined, the LVNC cohort displayed an increase in both QT intervals and positive LGE proportions. Furthermore, patients with lower FT3 levels in the T1 group experienced significantly higher incidence of malignant VT/VF, suggesting a potential link between low FT3 status and fibrosis induced arrhythmias in LVNC.

Third, FT3 may influence the onset of systemic thromboembolism in LVNC. TH deficiency can alter the coagulation pathway, but the clinical relevance remains uncertain. Previous investigations exploring coagulation in hypothyroidism have generated inconsistent conclusions, but more evidence suggested a tendency toward hypercoagulability [1, 36]. In the current study, LVNC patients with lower FT3 levels in the T1 group had a relatively higher proportion of cardiac thrombosis, however, the number of new thromboembolic events in the three groups were similar over the long-term follow-up. In order to clarify the underlying mechanism, multicentral prospective cohort studies with larger sample sizes will be needed.

In addition, FT3 can modulate genes that encode numerous important structural and functional proteins of the myocardium, including myosin heavy chains (encoded by MYH6 and MYH7), sodium/potassium-transporting ATPases, sarcoplasmic/endoplasmic reticulum calcium ATPase 2, and ryanodine channel, etc. Genetic analysis has indicated that mutations in the MYH7 and RYR2 genes might be linked to the pathogenesis of LVNC [37, 38], and the prevalence of MYH7 mutations is the highest among LVNC patients [17]. Coincidentally, both MYH7 and RYR2 genes are regulated by THs. Further exploration is warranted to determine whether low FT3 level will affect the development of LVNC by influencing its potential pathogenic genes.

Evidence from previous study has revealed that serum THs may not accurately reflect their levels in cardiac tissue [39]. Experimental and animal studies of heart failure showed that even though the concentration of serum T3 was not obviously decreased, impaired thyroid signaling and low cardiac local T3 were observed [40], indicating that serum THs levels might underestimate the cardiac low T3 status in heart failure. As was shown in our study, serum FT3 had an adverse influence on the cardiac function and prognosis in LVNC patients, even within the normal low range. RCS analysis illustrated that the incidence of all-cause mortality and MACE increased remarkably with the decrease of FT3 levels. Subgroup analysis suggested that lower concentration of FT3 was particularly detrimental in LVNC patients with LAD ≥ 40 mm or LVEF ≤ 35%. Large randomized controlled trials might be necessary in the future to investigate the effect of THs replacement on clinical prognosis in these particular subgroups of patients.

Several limitations should be considered when interpreting our data. Firstly, due to the retrospective nature of our cohort study, potential bias caused by incomplete follow-up could not be excluded. Encouragingly, the rates of missing data and follow-up failures were relatively low in the current study. In addition, our large LVNC cohort was recruited from a national-level cardiovascular center with extensive cardiac and thyroid function assessments at baseline, and all participants underwent evaluations according to consistent standard. Secondly, owing to the retrospective and observational design of the study, follow-up visits were not systematically scheduled. Very detailed information regarding the temporary and dynamic alterations in patients’ thyroid status was difficult to trace. Therefore, the focus of our study was to identify the association between serum FT3 levels at baseline and adverse cardiovascular outcomes in LVNC. Prospective cohort study should be conducted in the future to further explore the prognostic value of time-related changes of THs for LVNC. Thirdly, the assessment of thyroid function in our study was based on the serum TH levels instead of the myocardial tissue levels. As was mentioned previously, serum TH levels might not accurately indicate the TH levels in local cardiac tissue. As a result, it is essential to carry out further studies to determine the correlations between serum and myocardial TH levels in individuals with LVNC. Fourthly, information regarding presence of thyroid autoantibodies and thyroid ultrasonographic data were lacking. Therefore, some of the patients included in the study could have an unrecognized thyroid disease. Finally, even though our study has confirmed that adding FT3 to the pre-existing risk score for MACE [21] in LVNC improved its predictive performance, a causal relationship between FT3 levels and clinical outcomes is yet to be concluded given the observational study design. Randomized controlled trials with larger sample sizes in a multicenter setting are required to further validate our findings.

In conclusion, through the long-term investigation on a large cohort of morphologically diagnosed LVNC patients, we demonstrated that low FT3 level at baseline was an independent predictor for adverse cardiovascular outcomes, making FT3 a promising indicator for the risk stratification and clinical management in this specific population. Future studies on mechanisms are warranted to gain a better understanding of this underlying association, and our findings may encourage further exploration in this intriguing area.

### Supplementary Information

Below is the link to the electronic supplementary material.

Supplementary file1 (DOCX 34 KB)

## Data Availability

Research data of this study are not shared.
