# Supplementary material for: Long-term prognostic value of thyroid hormones in left ventricular noncompaction
Source: J Endocrinol Invest. 2024 Feb 15;47(9):2185–200. doi: 10.1007/s40618-024-02311-8 (PMC11369003; doi:10.1007/s40618-024-02311-8)
Supplement: Supplementary file 1 — Supplementary file1 (DOCX 34 KB) [file 40618_2024_2311_MOESM1_ESM.docx]

**Supplemental Table S1 Baseline characteristics of LVNC patients included and excluded**

|  | LVNC patients included  (N = 388) | LVNC patients excluded  (N = 48) | *P*-value |
| --- | --- | --- | --- |
| Age (years) | 43.5 ± 17.0 | 41.9 ± 13.8 | 0.542 |
| Male, *n* (%) | 253 (65.2%) | 30 (62.5%) | 0.711 |
| hypertension, *n* (%) | 105 (27.1%) | 12 (25.0%) | 0.761 |
| Hyperlipidemia, *n* (%) | 116 (29.9%) | 13 (27.1%) | 0.687 |
| diabetes mellitus, *n* (%) | 61 (15.7%) | 4 (8.3%) | 0.175 |
| NYHA class III/IV, *n* (%) | 206 (53.1%) | 23 (47.9%) | 0.498 |
| NSVT, *n* (%) | 101 (26.0%) | 10 (20.8%) | 0.436 |
| Cardiac Thrombosis, *n* (%) | 40 (10.3%) | 4 (8.3%) | 0.668 |
| LAD on TTE (mm) | 41.3 ± 8.4 | 42.2 ± 7.9 | 0.503 |
| LVEDD on tte (mm) | 61.1 ± 10.5 | 62.7 ± 8.2 | 0.287 |
| LVEF on tte (%) | 40.6 ± 14.6 | 39.6 ± 11.5 | 0.584 |

LVNC, left ventricular noncompaction; NYHA, New York Heart Association; NSVT, nonsustained ventricular tachycardia; TTE, transthoracic echocardiography; LAD, left atrial diameter; LVEDD, left ventricular end-diastolic diameter; and LVEF, left ventricular ejection fraction.

**Supplemental Table S2 Incidence of adverse clinical outcomes in the LVNC cohort**

| **Clinical Outcomes** | **Overall Patients**  **(N = 388)** |
| --- | --- |
|  |  |
| **Primary Outcomes** | 98 (25.3%) |
| Cardiovascular Mortality | 72 (18.6%) |
| Heart Transplantation | 28 (7.2%) |
| **Secondary Outcomes** |  |
| All-Cause Mortality | 75 (19.3%) |
| MACE | 130 (33.5%) |
| Cardiovascular Mortality | 72 (18.6%) |
| Heart Transplantation | 28 (7.2%) |
| CRT Implantation | 21 (5.4%) |
| Aborted SCD/VF | 15 (3.9%) |
| Sustained VT | 23 (5.9%) |
| appropriate ICD Therapy | 12 (3.1%) |
| Thromoembolism | 9 (2.3%) |

LVNC, left ventricular noncompaction; MACE, major adverse cardiovascular event; CRT, cardiac resynchronization therapy; SCD, sudden cardiac death; VF, ventricular fibrillation; VT, ventricular tachycardia; and ICD, implantable cardioverter defibrillator.

**Supplemental Table S3 Baseline characteristics of LVNC patients with or without NT-pro BNP levels**

|  | LVNC patients with NT-pro BNP  (N = 346) | LVNC patients without NT-pro BNP  (N = 42) | *P*-value |
| --- | --- | --- | --- |
| Age (years) | 43.8 ± 16.9 | 41.3 ± 17.4 | 0.368 |
| Male, *n* (%) | 224 (64.7%) | 29 (69.0%) | 0.580 |
| Cardiovascular Risk Factors*, *n* (%) | 210 (60.7%) | 22 (52.4%) | 0.299 |
| NYHA class III/IV, *n* (%) | 187 (54.0%) | 19 (45.2%) | 0.280 |
| FT3 (pg/ml) | 2.85 ± 0.51 | 2.92 ± 0.70 | 0.426 |
| FT4 (ng/dl) | 1.21 ± 0.19 | 1.23 ± 0.26 | 0.513 |
| TSH (mIU/l) | 1.91 (1.11-3.35) | 2.38 (1.22-3.42) | 0.266 |
| Albumin (g/L) | 43.4 (40.0-46.3) | 45.1 (41.9-47.3) | 0.052 |
| Alanine Aminotransferase (IU/L) | 22.0 (15.0-35.8) | 24.0 (16.0-46.5) | 0.401 |
| creatinine (μmol/L) | 82.8 (68.2-99.0) | 78.2 (63.8-91.1) | 0.167 |
| Glucose (mmol/L) | 5.07 (4.59-5.74) | 4.95 (4.50-5.32) | 0.075 |
| NSVT, *n* (%) | 93 (26.9%) | 8 (19.0%) | 0.275 |
| LAD on TTE (mm) | 41.7 ± 8.3 | 38.7 ± 9.0 | 0.046 |
| LVEDD on tte (mm) | 61.3 ± 10.6 | 59.1 ± 9.3 | 0.201 |
| LVEF on tte (%) | 40.2 ± 14.7 | 43.7 ± 14.2 | 0.144 |
| Cardiac Thrombosis, *n* (%) | 34 (9.8%) | 6 (14.3%) | 0.369 |

LVNC, left ventricular noncompaction; NT-pro BNP, N-terminal pro-brain natriuretic peptide; NYHA, New York Heart Association; FT3, free triiodothyronine; FT4, free thyroxine; TSH, thyrotropin; NSVT, nonsustained ventricular tachycardia; TTE, transthoracic echocardiography; LAD, left atrial diameter; LVEDD, left ventricular end-diastolic diameter; and LVEF, left ventricular ejection fraction. *Cardiovascular risk factors were defined as a composite of hypertension, dyslipidemia, diabetes mellitus, or smoking.

**Supplemental Table S4 Cox regression analysis on risk factors for the primary outcome in LVNC**

| **Variables**  **(N = 346)** | **Cardiovascular Mortality and/or Heart Transplantation (N = 84)** | | | | | | |
| --- | --- | --- | --- | --- | --- | --- | --- |
|  | **Univariable Analysis** | | | **Multivariable Analysis** | | | |
|  | **HR** | **95% CI** | ***P-*value** | | **HR** | **95% CI** | ***P-*value** |
| Age ^a^ | 1.010 | 0.997–1.024 | 0.126 | |  |  |  |
| Male ^a^ | 1.593 | 0.979–2.592 | 0.061 | |  |  |  |
| Cardiovascular Risk Factors ^a ,^* | 1.167 | 0.747–1.825 | 0.497 | |  |  |  |
| NYHA III or IV ^a^ | 3.365 | 1.997–5.671 | <0.001 | |  |  |  |
| FT3 ^a^ | 0.230 | 0.150–0.351 | <0.001 | | 0.428 | 0.270–0.679 | <0.001 |
| FT4 | 2.540 | 0.848–7.604 | 0.096 | |  |  |  |
| Ln TSH | 0.798 | 0.623–1.021 | 0.073 | |  |  |  |
| Ln NT-pro BNP ^a^ | 2.026 | 1.672–2.455 | <0.001 | | 1.527 | 1.214–1.920 | <0.001 |
| NSVT | 1.430 | 0.908–2.253 | 0.123 | |  |  |  |
| LAD (tte) ^a^ | 1.077 | 1.053–1.102 | <0.001 | | 1.042 | 1.014–1.071 | 0.003 |
| LVEDD (tte) ^a^ | 1.044 | 1.026–1.062 | <0.001 | |  |  |  |
| LVEF (tte) ^a^ | 0.946 | 0.929–0.963 | <0.001 | | 0.977 | 0.957–0.998 | 0.029 |
| Cardiac Thrombosis ^a^ | 1.968 | 1.065–3.638 | 0.031 | |  |  |  |

LVNC, left ventricular noncompaction; HR, hazard ratio; CI, confidence interval; NYHA, New York Heart Association; FT3, free triiodothyronine; FT4, free thyroxine; TSH, thyrotropin; NT-pro BNP, N-terminal pro-brain natriuretic peptide; NSVT, nonsustained ventricular tachycardia; TTE, transthoracic echocardiography; LAD, left atrial diameter; LVEDD, left ventricular end-diastolic diameter; and LVEF, left ventricular ejection fraction. * Cardiovascular risk factors were defined as a composite of hypertension, dyslipidemia, diabetes mellitus, or smoking. ^a^ Variables included in multivariable analysis on the cardiovascular mortality and/or heart transplantation.

**Supplemental Table S5 Cox regression analysis on risk factors for secondary outcomes in LVNC**

| **Variables**  **(N = 346)** | **All-cause Mortality (N = 62)** | | | | | | **MACE (N = 113)** | | | | | |
| --- | --- | --- | --- | --- | --- | --- | --- | --- | --- | --- | --- | --- |
|  | **Univariable Analysis** | | | **Multivariable Analysis** | | | **Univariable Analysis** | | | **Multivariable Analysis** | | |
|  | **HR** | **95% CI** | ***P-*value** | **HR** | **95% CI** | ***P-*value** | **HR** | **95% CI** | ***P-*value** | **HR** | **95% CI** | ***P-*value** |
| Age ^a, b^ | 1.039 | 1.021–1.057 | <0.001 | 1.029 | 1.012–1.046 | 0.001 | 1.018 | 1.007–1.030 | 0.002 |  |  |  |
| Male ^a, b^ | 1.884 | 1.039–3.419 | 0.037 |  |  |  | 1.071 | 0.722–1.587 | 0.734 |  |  |  |
| Cardiovascular Risk Factors *^, a, b^ | 1.813 | 1.037–3.169 | 0.037 |  |  |  | 1.262 | 0.856–1.860 | 0.240 |  |  |  |
| NYHA III or IV ^a, b^ | 2.630 | 1.469–4.711 | <0.001 |  |  |  | 2.812 | 1.833–4.314 | <0.001 |  |  |  |
| FT3 ^a, b^ | 0.255 | 0.158–0.412 | <0.001 | 0.462 | 0.269–0.795 | 0.005 | 0.348 | 0.242–0.500 | <0.001 | 0.587 | 0.396–0.870 | 0.008 |
| FT4 | 1.275 | 0.356–4.573 | 0.709 |  |  |  | 2.103 | 0.819–5.400 | 0.122 |  |  |  |
| Ln TSH ^a^ | 0.728 | 0.549–0.966 | 0.028 |  |  |  | 0.937 | 0.754–1.163 | 0.553 |  |  |  |
| Ln NT-pro BNP ^a, b^ | 1.947 | 1.553–2.441 | <0.001 | 1.775 | 1.372–2.298 | <0.001 | 1.656 | 1.428–1.921 | <0.001 | 1.317 | 1.099–1.579 | 0.003 |
| NSVT ^a^ | 2.095 | 1.259–3.489 | 0.004 |  |  |  | 1.459 | 0.985–2.163 | 0.060 |  |  |  |
| LAD (tte) ^a, b^ | 1.062 | 1.035–1.091 | <0.001 |  |  |  | 1.059 | 1.039–1.080 | <0.001 | 1.028 | 1.004–1.053 | 0.020 |
| LVEDD (tte) ^a, b^ | 1.037 | 1.016–1.059 | 0.001 |  |  |  | 1.038 | 1.022–1.054 | <0.001 |  |  |  |
| LVEF (tte) ^a, b^ | 0.955 | 0.936–0.975 | <0.001 |  |  |  | 0.956 | 0.942–0.970 | <0.001 | 0.979 | 0.962–0.996 | 0.015 |
| Cardiac Thrombosis ^b^ | 0.659 | 0.239–1.818 | 0.421 |  |  |  | 1.780 | 1.031–3.075 | 0.039 |  |  |  |

LVNC, left ventricular noncompaction; HR, hazard ratio; CI, confidence interval; NYHA, New York Heart Association; FT3, free triiodothyronine; FT4, free thyroxine; TSH, thyrotropin; NT-pro BNP, N-terminal pro-brain natriuretic peptide; NSVT, nonsustained ventricular tachycardia; TTE, transthoracic echocardiography; LAD, left atrial diameter; LVEDD, left ventricular end-diastolic diameter; and LVEF, left ventricular ejection fraction. * Cardiovascular risk factors were defined as a composite of hypertension, dyslipidemia, diabetes mellitus, or smoking. ^a^ variables included in the multivariable analysis on all-cause mortality. ^b^ variables included in the multivariable analysis on MACE.
